# Cartilage degradation is fully reversible in the presence of aggrecanase but not matrix metalloproteinase activity

**DOI:** 10.1186/ar2434

**Published:** 2008-05-30

**Authors:** Morten A Karsdal, Suzi H Madsen, Claus Christiansen, Kim Henriksen, Amanda J Fosang, Bodil C Sondergaard

**Affiliations:** 1 Nordic Bioscience A/S, Herlev Hovedgade 207, DK-2730 Herlev, Denmark; 2 University of Melbourne Department of Paediatrics and Murdoch Childrens Research Institute, Royal Children's Hospital, Flemington Road, Parkville, 3052, Melbourne, Victoria, Australia

## Abstract

**Introduction:**

Physiological and pathophysiological cartilage turnover may coexist in articular cartilage. The distinct enzymatic processes leading to irreversible cartilage damage, compared with those needed for continuous self-repair and regeneration, remain to be identified. We investigated the capacity of repair of chondrocytes by analyzing their ability to initiate an anabolic response subsequent to three different levels of catabolic stimulation.

**Methods:**

Cartilage degradation was induced by oncostatin M and tumour necrosis factor in articular cartilage explants for 7, 11, or 17 days. The catabolic period was followed by 2 weeks of anabolic stimulation (insulin growth factor-I). Cartilage formation was assessed by collagen type II formation (PIINP). Cartilage degradation was measured by matrix metalloproteinase (MMP) mediated type II collagen degradation (CTX-II), and MMP and aggrecanase mediated aggrecan degradation by detecting the ^342^FFGVG and ^374^ARGSV neoepitopes. Proteoglycan turnover, content, and localization were assessed by Alcian blue.

**Results:**

Catabolic stimulation resulted in increased levels of cartilage degradation, with maximal levels of ^374^ARGSV (20-fold induction), CTX-II (150-fold induction), and ^342^FFGVG (30-fold induction) (*P *< 0.01). Highly distinct protease activities were found with aggrecanase-mediated aggrecan degradation at early stages, whereas MMP-mediated aggrecan and collagen degradation occurred during later stages. Anabolic treatment increased proteoglycan content at all time points (maximally, 250%; *P *< 0.001). By histology, we found a complete replenishment of glycosaminoglycan at early time points and pericellular localization at an intermediate time point. In contrast, only significantly increased collagen type II formation (200%; *P *< 0.01) was observed at early time points.

**Conclusion:**

Cartilage degradation was completely reversible in the presence of high levels of aggrecanase-mediated aggrecan degradation. After induction of MMP-mediated aggrecan and collagen type II degradation, the chondrocytes had impaired repair capacity.

## Introduction

Osteoarthritis (OA) most likely results from altered biomechanical stress that leads to alterations in chondrocyte metabolism [1]. Cartilage turnover may be a more dynamic process than traditionally thought, with continuous remodeling of both the collagen and proteoglycan components of the articular matrix [2], although proteoglycans under physiological conditions may be more remodeled than collagens [3,4].

Cartilage turnover normally is maintained by a balance between catabolic and anabolic processes in which compensatory mechanisms in response to altered biomechanical stresses such as altered gait, weight distribution, or traumatic injury [1] ensure homeostasis in normal healthy individuals. This continuous turnover of cartilage may be an integrated part of reversible and physiologically important turnover. In contrast, a disturbance in the metabolism leading to an increase in the metabolic activity and activation of the pathological processes could lead to irreversible cartilage destruction [2,4]. Ideally, novel drugs designed to promote articular cartilage health should attenuate only pathological turnover and stimulate or maintain physiological turnover. However, at present, these processes have not been dissociated, most likely due to the lack of experimental systems and molecular tools for assessing cartilage turnover.

Studies in dogs have shown that proteoglycan loss from articular cartilage is reversible and that proteoglycan levels are restored after limited times of joint immobilization [4]. Furthermore, studies in animal models of cartilage degradation in which repair mechanisms can be studied, such as zymosan-induced arthritis and antigen-induced arthritis, demonstrated that cartilage damage was reversible only if the level of collagen II degradation was low [2]. However, these studies did not analyze aggrecanolysis mediated by the aggrecanases and matrix metalloproteinases (MMPs) separately or in detail.

Cartilage is composed predominantly of collagen type II (60% to 70% of dry weight) and proteoglycans (10% of dry weight); aggrecan is the most abundant proteoglycan in cartilage [5]. The key mediators of cartilage degradation include the MMPs and the closely related ADAMTS (a disintegrin and metalloproteinase with thrombospondin motifs) [6-12]. Aggrecan is degraded by both MMPs and ADAMTS, whereas collagen type II is degraded by MMPs, including MMP-1, -8, -13, and -14 [7,13-18]. These proteases release specific aggrecan or collagen II fragments that can be measured *in vitro *and *in vivo *[19]. Several of these molecular tools for assessing *in situ *cartilage degradation are new and have not been widely available. Only assays for measuring collagen type II degradation have been available in enzyme-linked immunosorbent assay (ELISA) formats [6,20-22]. Although assays for measuring sulphated glycosaminoglycans (S-GAGs) are available, these assays do not distinguish between synthesis and degradation of the proteoglycans [19]. Furthermore, they do not distinguish MMP-mediated degradation that generates DIPEN^341 ^and ^342^FFGVG fragments [23] from aggrecanase-mediated degradation that generates ITEGE^373 ^and ^374^ARGSV fragments [24]. Thus, these more specific markers of aggrecanolysis may further assist our understanding of cartilage turnover and repair.

Articular cartilage explants exposed to catabolic cytokines such as oncostatin M (OSM) and tumour necrosis factor (TNF) are useful *ex vivo *models of cartilage degradation with a high *in vivo *likeness, since the extracellular matrix is intact and contains all the regulators and natural structural components of articular cartilage [25]. In the present study, we investigated the enzymatic processes leading to irreversible cartilage destruction compared with continuous self-repair and regeneration with the aim of assessing when cartilage repair capacity was exhausted and reversibility was lost. We hypothesized that cartilage loss may be reversible if the catabolic period is short. We used OSM and TNF as catabolic stimulators to drive time- and concentration-dependent degradation of the cartilage matrix under standardized conditions [6]. Secondary to the catabolic induction, we investigated cartilage repair mechanisms after insulin growth factor (IGF)-I stimulation. IGF is a powerful anabolic growth factor that stimulates formation of type II collagen synthesis [26,27] and aggrecan synthesis [22,28] in cartilage explants *in vitro*.

## Materials and methods

### Reagents

All reagents were of analytical grade. The culture medium comprised 1:1 Dulbecco's modified Eagle's medium (DMEM) + Ham's F-12 with penicillin and streptomycin (all from Invitrogen Corporation, Carlsbad, CA, USA). Human recombinant OSM and recombinant human IGF were obtained from Sigma-Aldrich (Poole, UK), and human recombinant TNF-α was obtained from R&D Systems (Abingdon, UK).

### Tissue preparation

Bovine articular cartilage explants were carefully harvested by cutting with a scalpel the outermost layer of articular cartilage without adherent calcified cartilage from bovine heifer stifle joints between 1 and 1.5 years of age. The cartilage explants (12 to 14 mg) were washed three times in phosphate-buffered saline (PBS), placed in 96-well plates, incubated at 37°C, 5% CO_2_, and cultured under serum-free conditions in 200 μL of DMEM/F-12 containing cytokines in five replicates. As a control, articular cartilage explants and metabolically inactivated explants were cultured in DMEM/F-12. To deactivate the metabolism of the articular cartilage explants used for the 'metabolically inactive' (MI) condition (to investigate non-chondrocyte-mediated release of fragments), the explants were placed in cryo-tubes (Nunc, Roskilde, Denmark) and then frozen in liquid N_2 _and thawed at 37°C in a water bath for three repeated freeze-thaw cycles.

### Experimental design

**Figure 1:**
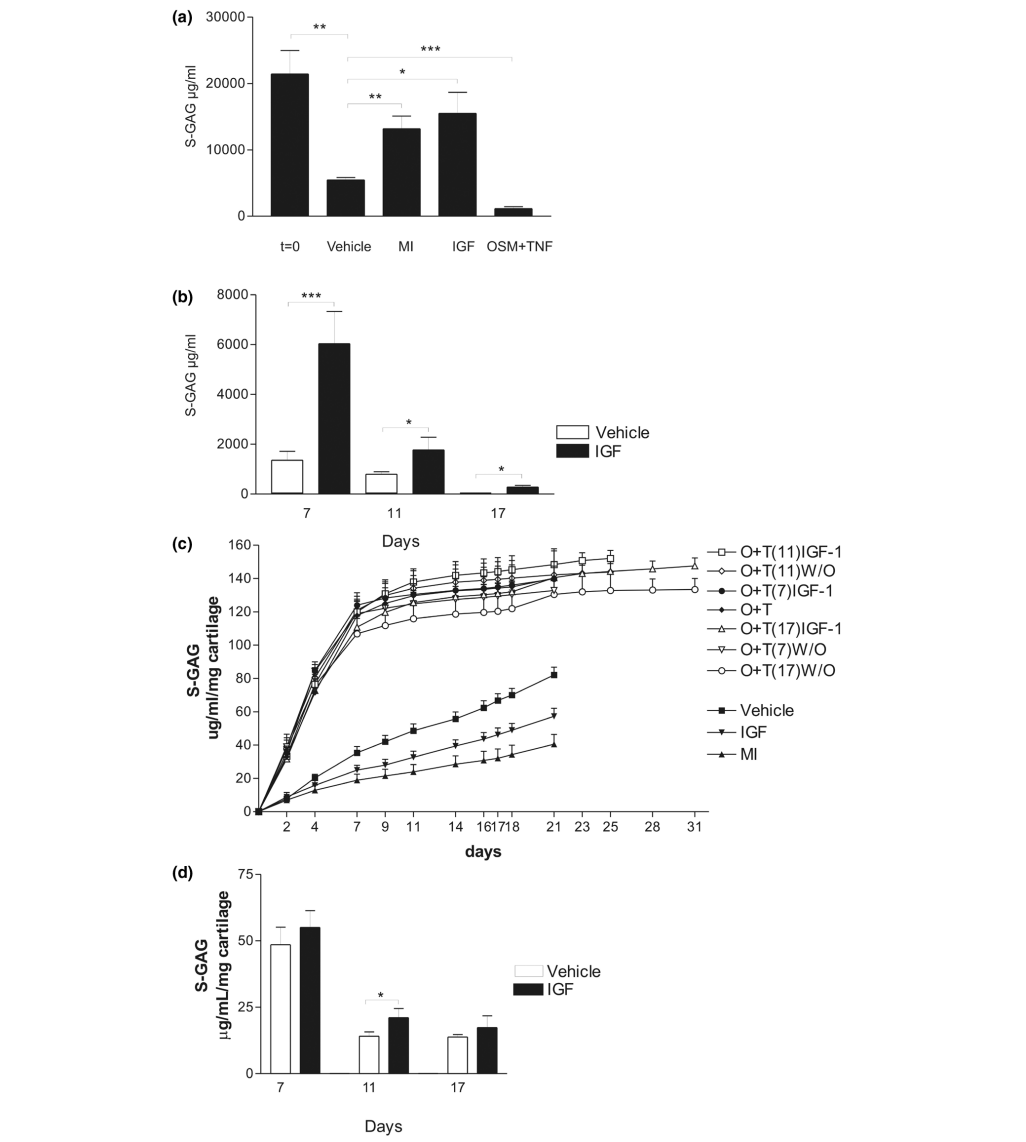
Quantification of aggrecan within the articular cartilage explants. The proteins of the cultured explants were extracted by liquid N_2 _pulverization. **(a) **Cartilage was extracted immediately after isolation (t = 0) or after culture for 21 days with vehicle, insulin growth factor (IGF), oncostatin M plus tumour necrosis factor (OSM + TNF), or metabolically inactive (MI) control for assessing passive physiochemical release. **(b) **Cartilage was extracted after the three different levels of cytokine treatment followed by an identical 14 days with either vehicle or IGF. IGF significantly stimulated proteoglycan content within the cartilage explants at all time points. **(c) **Quantification of sulphated glycosaminoglycan (S-GAG) from all treatments over the entire experimental period. S-GAG released from cartilage explants to the conditioned medium was quantified by the Alcian blue-binding assay. The curves represent the release at days when the conditioned medium was fully replaced, and the values were accumulated over the entire period. MI, metabolically inactive; O + T, oncostatin M plus tumour necrosis factor; W/O, without stimulation (vehicle control). **(d) **Quantification of S-GAG turnover 2 weeks after the catabolic induction. The aggrecan release in the identical 14-day period, with or without IGF stimulation following three different periods of catabolic stimulation, was measured by the Alcian blue-binding assay. The results show the accumulated release of S-GAG during the 2 weeks with anabolic stimulation (IGF) and without stimulation (vehicle). *P < 0.05, **P < 0.01, ***P < 0.001.

All cell cultures with bovine articular cartilage explants were approved by the local ethics committee. Articular cartilage explants were stimulated for 7, 11, or 17 days with the cytokines OSM (10 ng/mL) and TNF (20 ng/mL). Each catabolic period was followed by either (a) no stimulation or (b) (100 ng/mL) IGF stimulation for 2 weeks, resulting in total culture times of 21, 25, or 31 days (Figure 1c). Between the catabolic and anabolic phases, the explants were washed three times in PBS. On the last day of culture, samples from each treatment were either formaldehyde-fixed or snap-frozen. For other controls, additional samples were cultured for either 7, 11, or 17 days and treated without stimulation (vehicle), OSM + TNF, and IGF, and these samples were also formaldehyde-fixed and snap-frozen. Control treatments were analyzed in parallel on the same plate for vehicle, MI, (100 ng/mL) IGF, and OSM (10 ng/mL) + TNF (20 ng/mL) for 21 days and, on the last day, were formaldehyde-fixed or frozen for protein extraction. All treatment conditions were refreshed three times a week with freshly prepared medium plus stimulants. The conditioned medium was collected and stored at -20°C for further analysis. The use of MI cartilage as a control serves to control for the passive physical-chemical release of proteins and other molecules into the culture medium. Thereby, the difference between MI and vehicle is the cell-mediated release.

### Biochemical markers of cartilage degradation

#### a) Detection of CTX-II fragments

Crosslinked C-terminal neo-epitopes of type II collagen, CTX-II, is an MMP-mediated degradation fragment of collagen type II. CTX-II fragments were measured in the pre-clinical CartiLaps ELISA (IDS Ltd., Boldon, UK), which is an enzyme-linked immunoassay based on a mouse monoclonal antibody recognizing the six-amino acid epitope (EKGPDP) at the C-terminal telo-peptide of collagen type II. The assay can be used for measuring levels of CTX-II in conditioned media of explants cultures.

#### b) Detection of MMP-derived aggrecan fragment ^342^FFGVG-G2

Monoclonal antibody AF-28 recognizing the N-terminal neo-epitope generated by MMP cleavage at the amino acid sequence DIPEN^341^-^342^FFGVG localized in the inter-globular domain of aggrecan has been described previously [29] and manufactured by IDS Ltd., Boldon, UK. The ^342^FFGVG-G2 assay combines two monoclonal antibodies in a sandwich ELISA; the other antibody, F78, recognizes epitopes in the G1 and G2 globular domains of aggrecan [24].

#### c) Detection of aggrecanase-derived aggrecan fragment ^374^ARGSV

The ELISA detecting the aggrecanase-derived fragments of the N-terminal ^374^ARGSV combines two monoclonal antibodies in a sandwich ELISA system. The BC3 antibody (Abcam plc, Cambridge, UK) is used as the capturing antibody and the other antibody, F78, recognizes epitopes in the G1 and G2 globular domains of aggrecan [24]. In more detail, reagents and buffer were Rb × mouse IgG F(ab)_2 _from Chemicom International, Temecula, CA, USA and mouse monoclonal (BC-3) to Aggrecan ARGxx (ab3773) (Abcam plc). Stock standards were: Aggrecan from bovine articular cartilage (cat. no. A1960; Sigma-Aldrich) digested with ADAMTS-4. Recombinant Human ADAMTS-4 (Aggrecanase 1) (cat. no. CC1028; Millipore Corporation, Billerica, MA, USA). Peroxidase (POD)-conjugated F78 Ab (IDS Ltd., Bolton, UK). Normal Mouse Serum (Calbiochem, now part of EMD Biosciences, Inc., San Diego, CA, USA). Maxisorp plate cat. no. 438172, (Nunc). Coating solution: 10 mL of Na_2_CO_3 _buffer combined with 100 μL of 1 mg/mL of Rb × mouse IgG F(ab)_2_. Monoclonal buffer: 1:100 dilution of mouse monoclonal (BC-3) to aggrecan ARGS (ab3773) in PBS with bovine serum albumin and Tween (PBS-BTB) buffer. POD solution: 1:3,300 dilution of POD-conjugated F78 Ab dilution in PBS-BTB buffer containing 2.5% normal mouse serum. Standard dilution of ADAMTS-4 cleaved aggrecan, 12,500, 3,250, 3,125, 1,563, 781, 390, 195, and 0 ng/mL. Assay procedures: Maxisorp plates are coated with 100 μL of coating buffer overnight at 4°C without shaking. Washing five times, in PBS-BTB buffer. 100 μL of 1:100 dilution of mouse monoclonal (BC-3) to aggrecan ARGS antibody into each well, incubated for 1 hour at 20°C with 300 rpm shaking. Washing five times. 100 μL of diluted standards and samples into wells is added and incubated for 1 hour at 20°C with 300 rpm shaking. Washing five times. 100 μL of 300 ng/mL POD-conjugated F78 Ab containing 2.5% normal mouse serum is added and incubated for 1 hour at 20°C with 300 rpm shaking. Washing five times. 100 μL of TMB is added, incubated for 15 minutes at 20°C with 300 rpm shaking. After 15 minutes, the reaction is stopped with 100 μL of 0.18 M H_2_SO_4 _stopping solution. Optical density at 450 nm with 650 nm as reference is measured. The intra and inter-assay variations of the assay were 9.6% and 11.2%, respectively.

#### d) Detection of S-GAG

The concentration of S-GAG in conditioned medium and cartilage extracts was measured using the Alcian blue-binding assay (Euro-Diagnostica, Malmö, Sweden) according to the manufacturer's instructions.

### Biochemical markers of cartilage synthesis

Newly synthesized type II collagen was quantified as a marker of cartilage formation using a novel ELISA-based system [26]. This ELISA detects an internal amino acid sequence (GPQGPAGEQGPRGDR) in the pro-peptide from the N-terminal of collagen type II, the pre-clinical PIINP (IDS Ltd., Bolton, UK), and the assay was used for the assessment of cartilage formation from the conditioned medium according to the manufacturer's instructions.

### Extraction of the cartilage explants

The amount of S-GAG in the cartilage explants after termination of the culture was determined by extraction of the proteins by liquid N_2 _pulverization in quadruplicates. The explants were individually snap-frozen in liquid N_2 _and transferred to frozen stainless-steel pulverization aggregates and, by means of the Bessman tissue pulverizer (Spectrum Laboratories, Inc., Rancho Dominguez, CA, USA), were pulverized and solubilized in 10 volumes of ice-cold buffer: 50 mM Tris-HCl, pH 7.4, containing 0.1 M NaCl and 0.1% Triton X-100 with 1:100 protease inhibitor cocktail III (Calbiochem UK, now part of Merck, Darmstadt, Germany) and 20 μM GM6001 (Biomol International L.P., Plymouth Meeting, PA, USA), a general MMP inhibitor. Compared with that of the traditional procedure, this procedure, using guanidine extraction and papain digestion, results in 95% of the total yield of S-GAG. This approach was specifically chosen as it allows for measurement of the proteins and neo-epitopes. Papain or other digestions destroy peptide sequences. We detected neither pro-peptides nor neo-epitopes in normal unstimulated cartilage.

### Zymography

MMP-2 and MMP-9 expression and activity were determined by gelatinase zymography as described previously [6]. This technique allows for assessment of both pro-enzymes and active enzymes, which migrate differently according to their molecular weight during SDS-PAGE electrophoresis. This is important as all MMPs are synthesized as pro-enzymes, which then later are activated. The pro-enzyme is not activated under SDS-PAGE nor preparation but during overnight incubation in the activation buffer [6,30]. Briefly, 5 μL of the samples was loaded onto 7.5% SDS-polyacrylamide gels containing 0.5 mg/mL gelatin. After electrophoresis, the gels were incubated overnight at 37°C in 0.1% Triton X-100, 5 mM CaCl_2_, 1 mM ZnCl_2_, 3 mM NaN_3_, and 50 mM Tris pH 7.4 in a closed container, and then stained with coomassie blue, and finally destained, dried, and scanned for documentation.

### Histology

One cartilage explant from each treatment was taken out of culture on the appropriate day, fixed in formaldehyde, and processed for standard histology. Alcian blue was used to stain the proteoglycans in 5-μm sections. The sections were stained in a 1% solution of Alcian blue (Sigma-Aldrich) in 3% acetic acid (pH 2.5) for 30 minutes and rinsed in tap water for 2 minutes, and the nuclei were counterstained with Ehrlich's hematoxylin. The sections were dehydrated and mounted in DPX. Digital histographs were captured using an Olympus BX60 microscope with × 60 magnification and an Olympus C5050-zoom digital camera (Olympus, Tokyo, Japan).

### Statistics

All graphs show one representative experiment of at least three, each with at least four replicates. Mean values and standard error of the mean were calculated using GraphPad Prism (GraphPad Software, Inc., San Diego, CA, USA) and compared by the Student two-tailed unpaired *t *test of statistical significance assuming normal distribution. Asterisks indicate the significance levels (*P < 0.05, **P < 0.01, ***P < 0.001).

## Results

### OSM and TNF induce cartilage degradation, whereas IGF induces cartilage formation

A number of studies in different animal species have shown that OSM and TNF in combination induce cartilage degradation *in vitro*, in part through upregulation of both MMP and aggrecanase activities [6-11]. IGF induces cartilage formation with regard to both collagen type II and proteoglycan synthesis [26-28]. To investigate the repair and formation potential of distinct levels of pathological chondrocytes, we used these well-described cytokines to induce three different levels of catabolic activity followed by anabolic stimulation. The experiments were designed such that different levels of chondrocyte catabolism were induced (OSM + TNF) for 7, 11, and 17 days followed by identical lengths of culture with either IGF or vehicle (14 days) to investigate the capacity for repair.

### Anabolic stimulation indicates that cartilage degradation is completely reversible after short-term catabolic stimulation

The total content of proteoglycan retained in the articular cartilage explants was measured to determine whether aggrecan lost from the explants during the catabolic phase could be replaced during the subsequent anabolic phase. As seen in Figure 1a, IGF treatment increased total S-GAG content by approximately 125% compared with the vehicle control, in agreement with previous reports [28,31]. OSM + TNF activation alone resulted in more than 95% (*P *< 0.001) depletion of the proteoglycan content. Interestingly, articular cartilage cultured alone in the absence of cytokine induction lost 50% of proteoglycan compared with that to the MI control. compared to the levels of the negative control, metabolic inactive (MI). Compared with t = 0, the MI control lost 40% (*P *< 0.001) of total S-GAG content, suggesting a substantial physical-chemical diffusion from the culture compared with that of the cell-mediated release when comparing the vehicle with the MI control.

Chondrocytes in the articular cartilage explants exposed to the different levels of catabolic treatment responded differently to IGF treatment. IGF significantly increased the proteoglycan content in all the catabolically depleted explants (Figure 1b). Furthermore, we found that anabolic stimulation restored the S-GAG content in the explants completely when initiated after 7 days of catabolic treatment (comparing Figure 1a vehicle with Figure 1b IGF-stimulated), whereas at later stages only incomplete anabolic responses were obtained. These results indicate that cartilage degradation until day 7 is close to fully reversible, whereas proteoglycan depletion at days 11 and 17 is less reversible. One important limitation of the extraction experiments is that extracted S-GAG may be the result of both newly synthesized proteoglycans and the inhibition of loss of proteoglycans. However, as presented below, retained proteoglycans in the presence of IGF are positioned as circles around the chondrocytes, suggesting new synthesis, although this needs to be documented further.

Proteoglycan degradation, in addition to the extraction of proteoglycan from the cartilage plugs, can be measured by S-GAG release into the conditioned medium, although S-GAG release is more the result of turnover, in contrast to the MMP- and aggrecanase-generated neo-epitopes discussed previously. Figure 1c shows accumulated S-GAG release in the conditioned medium from all treatments. Stimulation with OSM and TNF resulted in substantially increased S-GAG release until day 7 compared with non-stimulated and MI explants. However, after the first 7 days of stimulation with OSM and TNF, there were negligible changes in S-GAG release, with or without subsequent anabolic stimulation, most likely because nearly all of the S-GAG was released by day 7. IGF stimulation, without previous catabolic stimulation, decreased S-GAG loss into the conditioned medium, consistent with its anabolic actions in cartilage.

To investigate the anabolic potential of chondrocytes following the catabolic periods, we accumulated the S-GAG levels released to the conditioned medium for the anabolic periods (days 7 to 21, 11 to 25, and 17 to 31) (that is, during the 14-day anabolic period subsequent to the catabolic insult). As seen in Figure 1d, when the different levels of pathologies were investigated, only small differences in S-GAG release were detected, in contrast to the measurements performed on the cartilage matrix itself.

### Collagen type II synthesis can be induced only after short-term degradation

**Figure 2:**
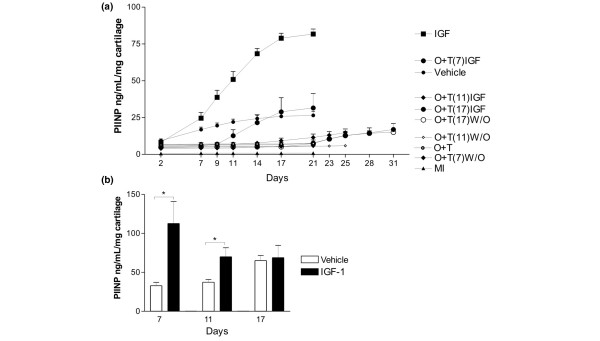
**Quantification of pro-peptides of collagen type II**. **(a) **Quantification of collagen type II synthesis from all treatments over the entire experimental period. Collagen type II synthesis in cartilage explants was measured by the concentration of N-terminal pro-peptides of type II collagen in the conditioned medium using the PIINP enzyme-linked immunosorbent assay (ELISA). The curves represent the release found at the specific day, where the conditioned medium was fully replaced, and the values were accumulated over the entire period. Vehicle control, metabolically inactive (MI), O + T, oncostatin M plus tumour necrosis factor. **(b) **Quantification of collagen type II formation 2 weeks after the catabolic induction. The collagen type II synthesis in the identical 14-day periods with or without IGF stimulation following the three different periods of catabolic stimulation was measured by the PIINP ELISA. The conditioned medium was fully replaced three times a week. The results show the accumulated release of collagen type II pro-peptide during the two weeks with anabolic stimulation (insulin growth factor, IGF) and without stimulation (vehicle). IGF-I significantly induced collagen type II formation at low and intermediate catabolic insult, but not at maximal insult. *P < 0.05. PIINP, N-terminal pro-peptide of pro-collagen type II.

To further investigate the anabolic response of chondrocytes to IGF after different levels of catabolic stimuli, we measured the release of the N-terminal pro-peptide of pro-collagen type II, PIINP, as a marker of collagen type II synthesis [26]. As expected, metabolically inactivated explants showed no type II collagen synthesis, whereas IGF stimulation throughout the culture period resulted in a 4-fold induction of collagen type II synthesis compared with the vehicle control (Figure 2a). In addition, the OSM + TNF-stimulated explants did not synthesize or release collagen II pro-peptides.

To investigate the anabolic potential of chondrocytes following the catabolic periods, we accumulated the PIINP levels released to the conditioned medium for the anabolic periods (days 7 to 21, 11 to 25, and 17 to 31) (that, is during the 14-day anabolic period subsequent to the catabolic insult). After 7 days of catabolic stimulation, collagen type II synthesis increased in response to IGF treatment. However, we observed a lower level of IGF-induced collagen II synthesis after 11 days of cytokine treatment and no IGF-induced collagen II synthesis after 17 days of cytokine treatment (Figure 2b).

Interestingly, under the current culture conditions, the cartilage did not lose the IGF-I responsiveness during prolonged culture periods. When IGF-I was added after 7, 11, or 17 days of culture, a similar induction of cartilage synthesis was observed (data not shown). In addition, these data suggest that cartilage has low levels of continuous collagen type II formation measured by the PIINP assay, however these levels could potently be stimulated by IGF-I exposure.

To further investigate the amount of PIINP that was retained in the cartilage compared with that which was released, we extracted articular cartilage either non-stimulated or stimulated with either catabolic or anabolic stimulation. We are not able to detect PIINP under any conditions (data not shown). These data suggest that n-telo-peptides of pro-collagen type II under the current culture condition are almost exclusively released during synthesis and thereby may be valid markers for collagen type II formation. These data further support our hypothesis that cartilage loss is reversible if the catabolic stimulation is short. Similarly, the potential for reversing cartilage degradation diminishes if cytokine treatment is extensive.

### Assessment of aggrecanase- and MMP-mediated cartilage degradation indicates that loss of repair mechanisms occurs after induction of MMP activity

**Figure 3:**
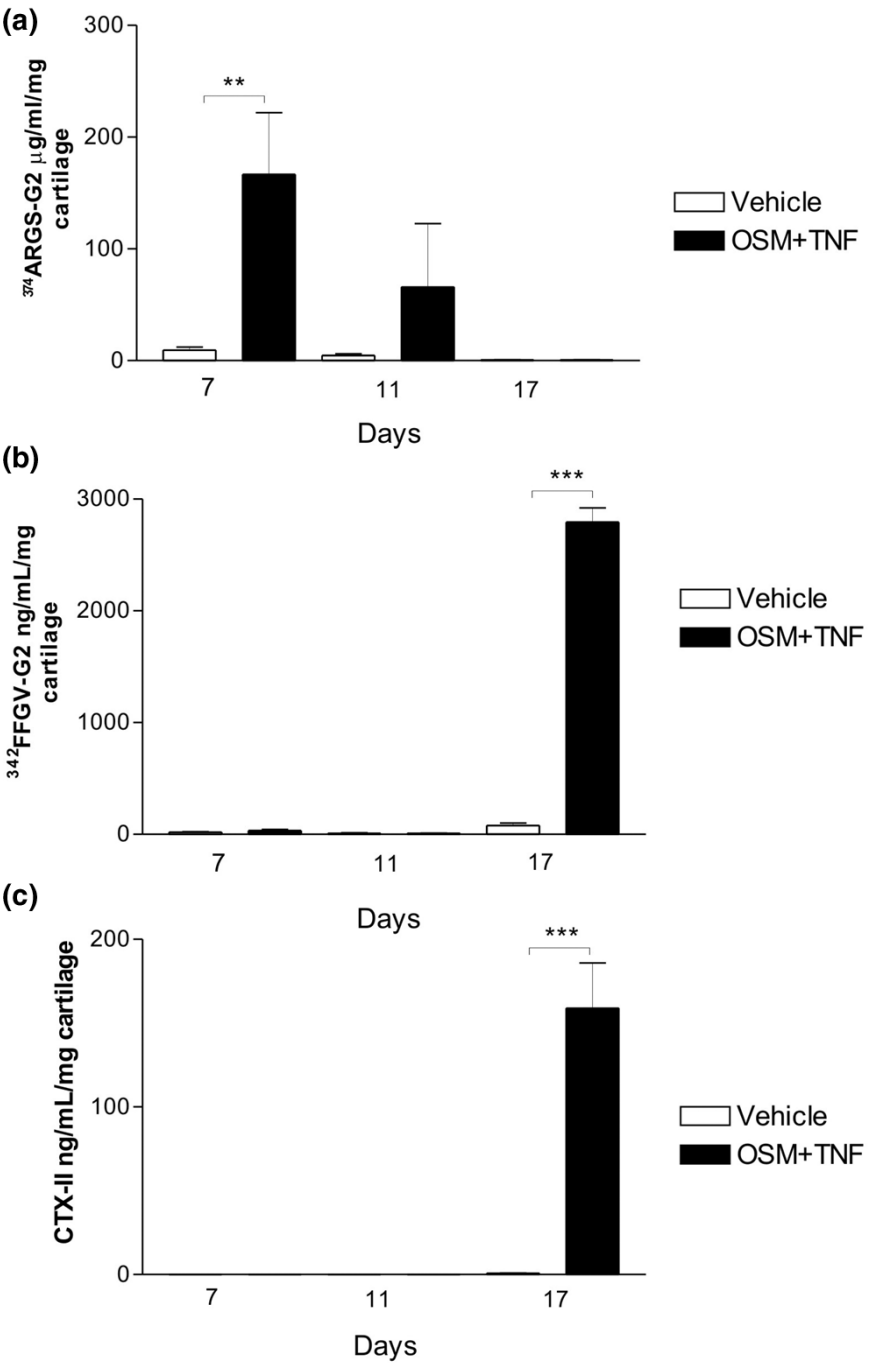
Quantification of aggrecan and collagen degradation products at days 7, 11, and 17. Articular cartilage explants were cultured in the presence or absence of oncostatin M plus tumour necrosis factor (OSM + TNF). Conditioned medium was collected at days 7, 11, and 17. **(a) **Aggrecanase-mediated aggrecan degradation was measured by the ^374^ARGSV-G2 enzyme-linked immunosorbent assay (ELISA), **(b) **matrix metalloproteinase (MMP)-mediated aggrecan degradation was quantified by the ^342^FFGVG-G2 ELISA, and **(c) **MMP-mediated collagen type II degradation was quantified in the CTX-II ELISA. **P < 0.01, ***P < 0.001. CTX-II, crosslinked C-terminal neo-epitopes of type II collagen.

To further characterize the molecular mechanism underlying the loss of repair capacity, we measured levels of the catabolic biomarkers ^374^ARGSV, ^342^FFGVG, and CTX-II after the individual catabolic treatments. We found that OSM + TNF-stimulated degradation, mediated by aggrecanases and measured using the ^374^ARGSV-G2 assay, was high at day 7, intermediate at day 10, and almost absent at day 17 (Figure 3a). This is consistent with the S-GAG release data showing that the majority of S-GAGs are released at the early stages of catabolic stimulation. The levels of the MMP-generated fragment ^342^FFGVG-G2 showed that MMP-mediated aggrecan was undetectable at days 7 and 10 and high at day 17 (Figure 3b). The high aggrecanase activity at the early stages of culture may mask the MMP-mediated aggrecan epitope (^342^FFGVG-G2) by further processing in generating the aggrecanase (^374^ARGSV) site; however, Fosang and colleagues [32] have found that further processing of ^342^FFGVG to generate ^374^ARGSV cannot occur, at least not *in vitro*. High levels of MMP activity should have generated CTX-II fragments that are not further processed by other proteases, suggesting that MMP activity is present only at a lower level at early culture time points. This was verified by the use of a fluorescence substrate technique, in which MMP levels were detectable only in the presence of catabolic stimulation and only at late time points (data not shown), which correlate well with previous findings, documenting extensive MMP activities at later stages of catabolic induction but not at early stages [6,9,32]. Interestingly, most S-GAG is released at earlier time points than the ^342^FFGVG release, indicating that aggrecan loss is due primarily to aggrecanase activity, but later, aggrecanolysis shifts to an MMP-mediated degradation mode. Finally, we found that the release of the collagen type II degradation fragment CTX-II (Figure 3c) occurred with a pattern similar to that of ^342^FFGVG (Figure 3b), consistent with the fact that the CTX-II fragment is MMP-generated [33].

In summary, these data appear to mimic cartilage degradation in arthritis where aggrecanase activity on aggrecan precedes MMP mediated aggrecan degradation that is subsequently followed by MMP degradation of collagen, which has been reported with various techniques from other labs [32]. In addition, these data show that there is a positive correlation between MMP activity (evidenced by the ^342^FFGVG-G2 and CTX biochemical markers) and the inability of cytokine-treated chondrocytes to initiate and/or maintain anabolic activity.

### Switching to anabolic stimulation after short-term catabolic stimulation can reduce MMP activity

**Figure 4:**
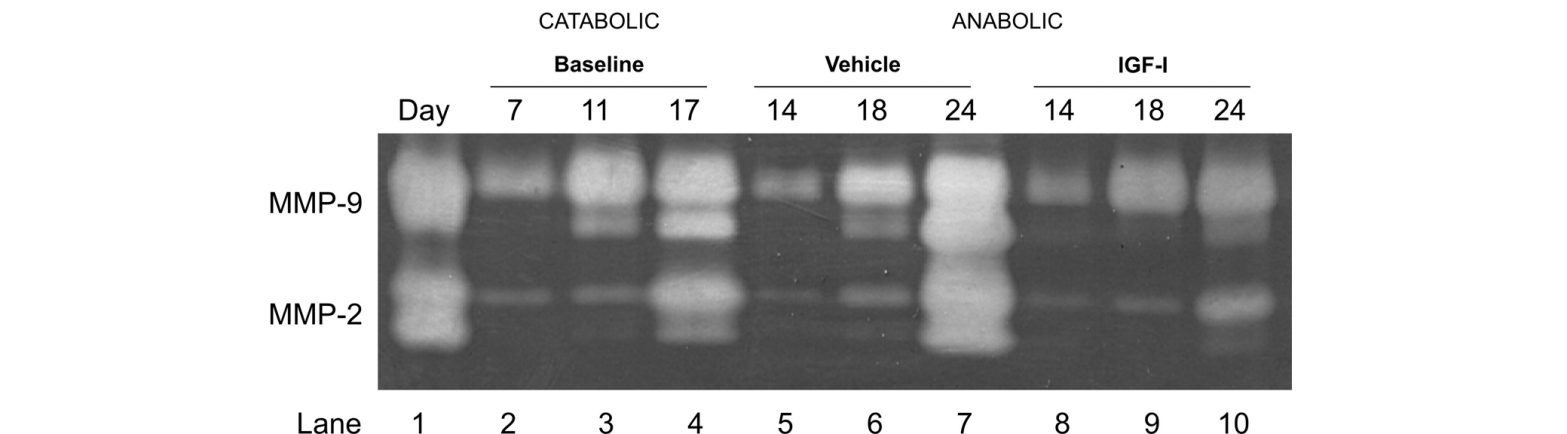
Gelatinase activity is attenuated but not abrogated during insulin growth factor (IGF) stimulation. Gelatinase activity in conditioned medium from bovine articular cartilage explants was investigated by zymography. Lane 1 shows standards for matrix metalloproteinase (MMP)-9 + MMP-2. Conditioned medium at the end of each catabolic culture period (7, 11, or 17 days) was used as a reference (lanes 2 to 4). Conditioned medium from cultures treated with IGF (lanes 8 to 10) or vehicle (lanes 5 to 7) 7 days after the catabolic period was analyzed. Compared with vehicle and baseline measurements, IGF only attenuated MMP production and activation.

To further investigate the protease levels during anabolic and catabolic phases of chondrocyte stimulation, we measured MMP activity by gelatine zymography (Figure 4). The ^342^FFGVG-G2 and CTX-II peptides are generated by an array of MMPs, of which MMP-2 and MMP-9 are only a subset. On other occasions, the presence of these gelatinases has been a valid indication of total MMP activity and thereby the catabolic potential of the culture [6]. Gelatinase activity at 7, 11, and 17 days after catabolic treatment was compared with gelatinase activity after 7 days of IGF treatment, corresponding to the middle of the anabolic stimulation period. We found that gelatinase activity and expression were attenuated by IGF, but not completely reversed, compared with gelatinase activity after 7 days with vehicle alone (Figure 4). The results with samples analyzed after 14 days of IGF or vehicle were similar to those for 7 days of IGF or vehicle (data not shown). The presence of active MMP-2 and MMP-9 at days 11 to 17 corresponds to the period when high levels of ^342^FFGVG-G2 and CTX-II are detected in Figures 3a and 3c. These data also indicate that, even in the presence of substantial MMP activity, chondrocytes are able to synthesize new aggrecan and proteoglycans (Figure 1b), but not collagen type II (Figure 2b).

### Proteoglycan staining confirms the pattern of reversibility

**Figure 5:**
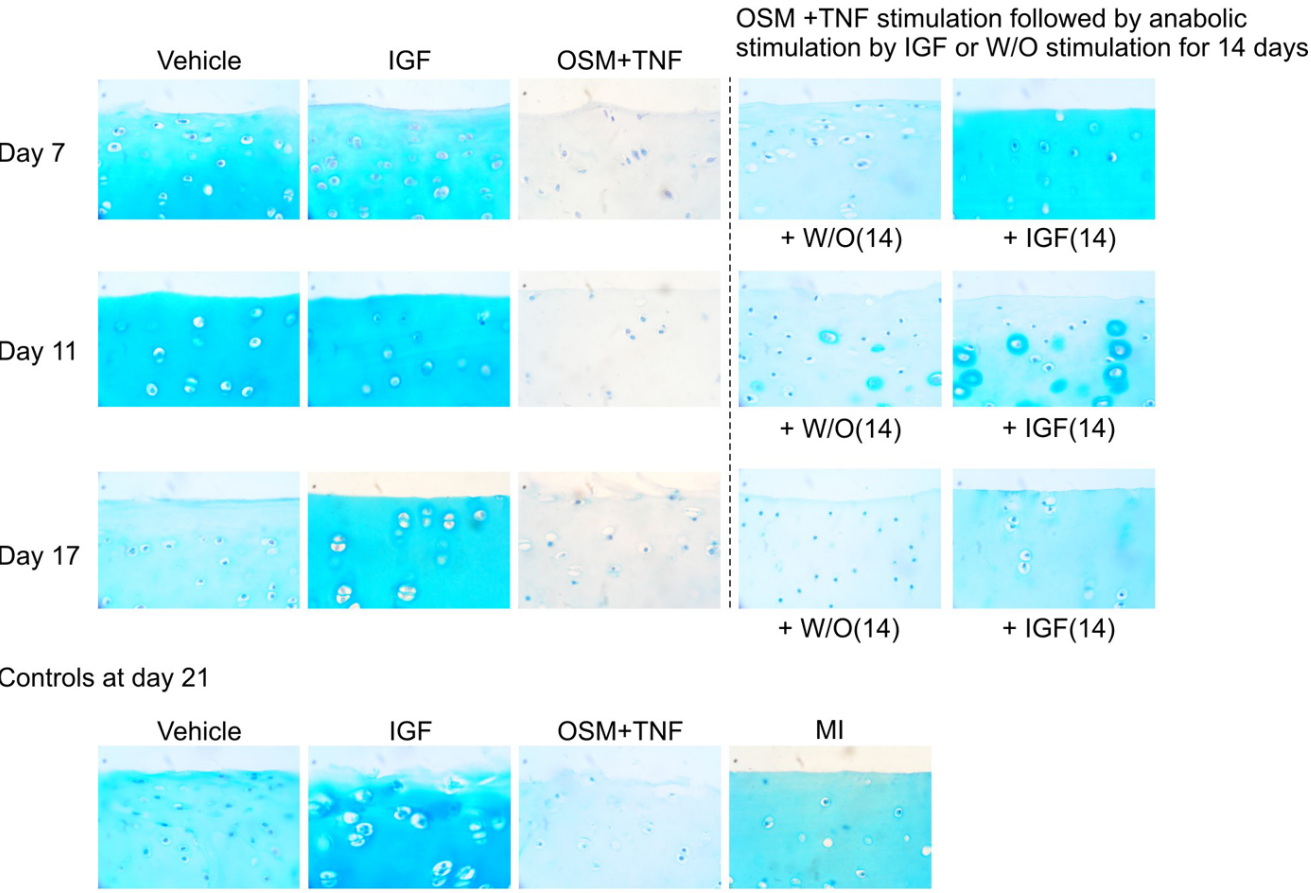
Insulin growth factor (IGF) stimulates local replenishment of cartilage. Articular cartilage explants were cultured with either oncostatin M plus tumour necrosis factor (OSM + TNF) or vehicle for 7, 11, and 17 days. Subsequently, cartilage explants were paraffin-embedded and stained for aggrecan content as described in Materials and methods. Aggrecan is completely depleted from the tissue at 7, 11, and 17 days. Other cultures were treated with either OSM + TNF or vehicle for 7, 11, and 17 days followed by stimulation with either IGF or vehicle control for 14 days. Subsequently, cartilage explants were paraffin-embedded and stained for aggrecan content as described in Materials and methods. As a control experiment, articular cartilage explants were cultured for 21 days with vehicle, OSM + TNF, IGF, or metabolically inactive (MI) control for 21 days as controls (lower panel). W/O, without stimulation.

To visualize the repair enhanced by IGF treatment, cultured cartilage was harvested at different time points. Proteoglycans in the cartilage were visualized using Alcian blue staining, the same dye used in the S-GAG assay. The control articular cartilage explants (shown in the bottom row of Figure 5) were cultured for 21 days with vehicle, OSM + TNF, IGF, or MI control for 21 days. In complete agreement with the S-GAG quantifications in Figure 2, IGF increased whereas OSM + TNF decreased GAG content compared with vehicle. MI control contained more GAG compared with vehicle as the cell-mediated loss of proteoglycan content was abrogated. With regard to the dynamics in the reversibility experiments presented in the upper panels, the vehicle control explants gradually lost S-GAG content over time, whereas the explants treated with IGF maintained the S-GAGs, even after 17 days in culture. OSM + TNF treatment depleted proteoglycans from the matrix maximally by day 7, consistent with the results in Figure 1c which show that S-GAG release into the medium is also maximal by day 7. Treatment with IGF stimulated GAG synthesis in the explants that were treated with cytokines for 7 and 11 days, but not for 17 days. IGF treatment of explants after 7 days of catabolic stimuli restored proteoglycan content throughout the entire cartilage matrix. IGF treatment after 11 days of catabolic treatment showed new proteoglycan synthesis around chondrocytes, indicative of repair. There was also evidence of repair in the absence of IGF treatment after 11 days of catabolic stimuli; however, the repair was substantially improved in the presence of IGF. Chondrocytes treated with catabolic cytokines for 17 days reinitiated, only to a very minor extent, proteoglycan synthesis in the presence of IGF compared with that of vehicle. These data support the idea that cartilage degradation may be more reversible before induction of MMP activity.

## Discussion

OA is the most common degenerative disease of the joints [34,35] and this multifactorial and diverse disease is characterized by increased activity of at least two groups of enzymes, the MMPs and the ADAMTS, which mediate the degradation of the type II collagen and aggrecan-containing matrix [19]. However, the molecular sequence of events leading to irreversible damage and the level of cartilage destruction at which the damage becomes irreversible remain to be investigated. With the recent development of assays for the detection of type II collagen synthesis *ex vivo*, as well as both MMP- and aggrecanase-mediated degradation of aggrecan [24], cartilage turnover can be assessed in more molecular detail.

By using a combination of OSM and TNF (which is known to induce pathological degradation [6]) and anabolic stimulation by IGF (which is a known powerful anabolic growth factor for chondrocytes [26]), we assessed the anabolic potential of the three stages of pathologically activated chondrocytes. We found that once MMP-mediated degradation was in progress, the capacity for repair was completely lost with regard to collagen type II synthesis, whereas proteoglycan synthesis was strongly attenuated. In contrast, at the time of maximal aggrecanase activity, the proteoglycan loss was fully reversible. These findings correlate well with previous *in vivo *studies indicating that aggrecan loss was reversible as long as the progression was not too advanced [2,4,36]. In further support of these findings are studies in inflammatory arthritis models which indicated that only low levels of type II collagen degradation could be reversed [2,4,36]. The present data further support these findings, and demonstrate that even in this simple ex vivo system, the molecular mechanism of action underlying the irreversible degeneration of cartilage involves the induction of MMP activities, whereas the aggrecanases mainly are involve in reversible processes.

To examine whether the anabolic growth factor IGF could affect protease activities, we investigated MMP expression at the end of the catabolic stimulation and after the anabolic period (Figure 4). Surprisingly, anabolic induction after the catabolic period did not result in a complete abrogation of MMP activity, but only a reduction as seen in Figure 4. This suggests that, even in the presence of increased protease activities, chondrocytes are able to start making new matrix. The *ex vivo *studies presented here indicate that cartilage degradation may be more reversible than previously thought.

Studies have elucidated that chondrocytes in a series of complicated events involving gene transcription lose their IGF responsiveness and thereby potentially lose their repair capacity, in part through nitric oxide exposure and upregulation of SOCS3 (suppressor of cytokine signaling 3) [27,37,38]. This might contribute in part to the loss of reversibility, as reversibility in the current studies was investigated as IGF responsiveness. The current studies showed complete reversibility after 7 days of cytokine treatment and showed attempted repair (aggrecan pericellular staining after 14 days), though under different experimental conditions. Even after extensive catabolic insult, some proteoglycan synthesis was seen when exposed to IGF-I. Interestingly, the articular cartilage under the current culture conditions did not lose its IGF-I responsiveness. When articular cartilage was exposed to IGF stimulation at days 7, 11, and 17 in the absence of catabolic stimulation, similar inductions of PIINP syntheses were observed (data not shown).

With regard to the possible continuous turnover of collagen type II and proteoglycans in the articular cartilage matrix, the current experiments may provide some additional information. We observed a continuous synthesis of collagen type II even in non-stimulated conditions (Figure 2a). Thus, these data further support the notion that both collagen type II and proteoglycans are continuously turned over in the articular matrix, although the proteoglycan turnover may be superior to that of the collagen turnover. In the current experiments, this is best visualized by the nanogram quantities of pro-collagen epitopes compared with the microgram quantities of S-GAG and the aggrecanase-generated epitopes of aggrecan, ARGS-G2. These data are in agreement with those of previous investigators concluding that aggrecanases are the major mediators of aggrecan turnover [12,39] and that proteoglycans are remodeled to a higher degree compared with that of collagen type II [40-42].

The current experiments have measured the release of degradation products from the articular matrix as markers of protease activities. The sequential timing, coordination, individual roles, and the interactions between MMP and aggrecanase activities are highly researched topics that are only beginning to be partly understood. The data do not provide the complete answer but hopefully add a piece of the highly complicated puzzle. Most interestingly, aggrecanase-mediated aggrecan degradation was virtually absent at the end of the study period; instead, the release of MMP-derived aggrecan fragments was detected at this time. We have verified that there indeed are high levels of aggrecanase activity present at later stages of the culture period (data not shown). The results in Figure 3 suggest that there is a population of aggrecan that is resistant to aggrecanase cleavage. This population 'survives' high levels of aggrecanase activity for up 17 days but is then cleaved by MMPs. This separate pool of aggrecan molecules that have a different protein degradation profile needs to be investigated in more molecular detail and may allow for further understanding of the molecular events leading to cartilage destruction. Many alternative hypotheses and conditions, including but not limited to the following, need to be investigated: (a) whether the aggrecanases have been processed, altering their substrate specificity (possibly by MMPs), (b) whether the lack of aggrecanase-mediated aggrecanolysis is due to limited availability of aggrecan for ADAMTS-mediated turnover (possibly due to processing at the cell surface of newly synthesized aggrecan molecules by membrane-type MMPs), and (c) whether the extensive aggrecanase activity early in the cultures masks the MMP-generated fragments of aggrecan, which in theory could be possible as aggrecanase activity would shed the MMP site from the aggrecan molecule. With regard to whether aggrecanase activities mask the MMP-generated extracellular matrix fragments of aggrecan, additional information may be found in the present data. If aggrecanase activities should have masked the MMP-mediated activity on aggrecan as a consequence of high levels of MMP activity, the MMP-generated collagen type II epitope CTX-II should have been generated, as the CTX-II epitope is a promiscuous site generated by most MMPs [6,33]. The absence of both CTX-II and the MMP-mediated aggrecan fragment at the early culture days suggests lower levels of MMP activity at these time points compared with those of later time points. The low level of MMP activity early in the cultures compared with the extensive activity later under catabolic induction was verified by the use of a fluorescence substrate (data not shown), which was in complete agreement with previous findings using other techniques [6].

This and other studies begin to suggest that OA may be approached differently depending on the level of disease progression, in which each stage would require different intervention strategies. Our studies suggest that interventions of OA by anabolic therapies may be useful. These possible anabolic strategies should be able, at best, to regenerate cartilage or at least to replenish lost aggrecan in the articular cartilage. Since the method developed in this study corresponds well to the situations seen in vivo, with respect to generation and regeneration of cartilage damage, we speculate that it should be implemented for testing the chondro-anabolic effect of different drugs.

This is based, in particular, on the fact that damaged cartilage or cytokine-primed cartilage responds differently than normal cartilage [43] and has less of an anabolic response [37]. The latter study indicated that, in particular, the anabolic response in chondrocytes to IGF was dependent on the cytokine milieu [27]. Therefore, if OA is diagnosed sufficiently early, more cartilage than traditionally thought may be regenerated or preserved. The current data indicate that chondrocytes are responsive to anabolic stimulation even at significant MMP activity levels and that aggrecanase activities have very little effect, if any, on the level of repair capacity.

The current study has some important limitations, which include the use of young bovine cartilage and the use of the synchronous cultures. The synchronous induction of cartilage degradation may be different from that seen overall in a weight-bearing joint, although the same processes are entailed. Furthermore, the current experiments were conducted under *ex vivo *conditions, in which cartilage is cultured under non-weight-bearing conditions, in which cutting of the cartilage may induce alternative metabolism. This may influence the cartilage metabolism and thereby allow for skewed interpretations of the turnover compared with that of the *in vivo *conditions.

Our results show that, under the influence of anabolic stimuli, cartilage explants depleted of aggrecan by aggrecanases can restore their aggrecan content provided that the catabolic stimulation has not been too severe. We have yet to explore the precise mechanism of how this is achieved, but it is likely to reflect the imbalance between aggrecan synthesis/retention and aggrecan degradation. The balance is more likely to be tipped in favour of retention after a short catabolic period than a long one. When chondrocytes that have received the short cytokine treatment commence new matrix synthesis, they might do so more effectively because the cells are 'healthier' than cells exposed to a long cytokine treatment. The results show that chondrocytes exposed to both 7 days and 11 days of catabolic treatment are able to compensate by self-repair, but the rate at which repair is initiated and then continued is less in explants receiving the longer treatment. Very little proteoglycan synthesis was possible in explants exposed to the longest treatment (17 days), suggesting that the exposure of chondrocytes in these explants was chronic and interfered substantially with normal chondrocyte function.

## Conclusion

We have developed a model and molecular tools that allowed us to investigate the repair-capacity potential of pathologically activated chondrocytes. We found that once MMP-mediated type II collagen and aggrecan degradation was induced, the reversibility was lost as determined by collagen type II synthesis, whereas proteoglycan synthesis was strongly attenuated. Interestingly, even in the presence of extensive aggrecanase activities, cartilage degradation seems completely reversible.

## Abbreviations

ADAMTS = a disintegrin and metalloproteinase with thrombospondin motifs; CTX-II = crosslinked C-terminal neo-epitopes of type II collagen; DMEM = Dulbecco's modified Eagle's medium; ELISA = enzyme-linked immunosorbent assay; GAG = glycosaminoglycan; IGF = insulin growth factor; MI = metabolically inactive; MMP = matrix metalloproteinase; OA = osteoarthritis; OSM = oncostatin M; PBS = phosphate-buffered saline; PBS-BTB = phosphate-buffered saline with bovine serum albumin and Tween; PIINP = N-terminal pro-peptide of pro-collagen type II; S-GAG = sulphated glycosaminoglycan; TNF = tumour necrosis factor.

## Competing interests

MAK and CC are stockholders of Nordic Bioscience. AJF declares that she has no competing interests. All other authors are full-time employees of Nordic Bioscience.

## Authors' contributions

MAK designed the study, wrote the manuscript, and participated in all parts of the experiments. AJF and CC critically reviewed the manuscript, provided expert advice, and participated in the drafting of the manuscript. SHM, KH, and BCS carried out cartilage explants cultures, histology, proteoglycan extraction, and measurements. All authors read and approved the final manuscript.
